# Rice: The First Crop Genome

**DOI:** 10.1186/s12284-016-0087-4

**Published:** 2016-03-22

**Authors:** Scott A. Jackson

**Affiliations:** Center for Applied Genetic Technologies, University of Georgia, Athens, GA 30621 USA

**Keywords:** Rice, Genome Sequencing, Translation, Gene Mapping

## Abstract

Rice was the first sequenced crop genome, paving the way for the sequencing of additional and more complicated crop genomes. The impact that the genome sequence made on rice genetics and breeding research was immediate, as evidence by citations and DNA marker use. The impact on other crop genomes was evident too, particularly for those within the grass family. As we celebrate 10 years since the completion of the rice genome sequence, we look forward to new empowering tool sets that will further revolutionize research in rice genetics and breeding and result in varieties that will continue to feed a growing population.

## Introduction and Review: Rice as a Model Genome

### The Rice Genome

The rice genome was one of the few truly multinational plant genome projects, one with flags planted in chromosomes (Eckardt 2000)—Japan, chromosomes 1, 6, 7 & 8; US, chromosomes 3 & 10; China, chromosome 4; France, chromosome 12; Taiwan, chromosome 5; etc.—like early explorers claiming new territories. Arabidopsis had been sequenced and published in 2000 (Arabidopsis Genome I 2000) and rice, nearly four times the size, was next. These were heady days for plant genome researchers, a discipline still in its infancy. Researchers jockeyed to be part of the project and, like every good collaborative project, multitudinous meetings were held to plan, to execute and, finally, to celebrate the rice genome project. These meetings were often tense—deciding how chromosomes were distributed, discussing progress, or lack thereof—and the omnipresent funding agency representatives hovered about ensuring that their agencies funds were well spent, the most diminutive of whom could elicit fear in the most seasoned genome researcher.

Rice was one of the last clone-by-clone, Sanger-sequenced genomes. That is, BAC/PAC clones were sequentially selected for sequencing, independently assembled and then stitched together to form pseudo-chromosomes. One impact that the rice genome had, even in the midst of sequencing, was the first completely sequenced, complex eukaryotic centromere on chromosome 8 (Nagaki et al. 2004), which are usually gaps in genome sequences as they are highly repetitive regions of the genome. This was possible due to its diminutive size, ~64 kbp of satellite repeats, even compared to even Arabidopsis centromeres. Thus, rice has been a model for studies of centromere structure and function. This was possible due to cytogenetic analysis of rice showing the diminutive centromere 8. Interestingly, if the US had heeded cytogenetic descriptions they may not have chosen chromosome 9 as a sequencing target as one arm is highly heterochromatic, full of repetitive DNA sequences, which complicated physical mapping and sequencing of that chromosome arm.

The public rice genome, which took advantage of whole genome shotgun sequenced genomes made available from Monsanto in 2000 and Syngenta in 2002 (Goff et al. 2002), was published in 2006 (International Rice Genome Sequencing P 2005) after which the arduous task of annotation took place. Rice was one of the few genomes to have competing annotations (Ohyanagi et al. 2006; Ouyang et al. 2007), which created some confusion in the community but was eventually resolved with a single unified annotation (Kawahara et al. 2013).

### Rice as a Model Cereal Genome

One of the initial motivators for sequencing rice, besides the relatively small genome size, was that it could be used as a model for other cereal crops with larger genomes, such as maize and wheat. This was predicated somewhat on rice’s small genome size and the realization from molecular mapping, e.g. RFLPs, of conserved markers and marker order. Early work had shown conserved synteny between sorghum and maize (Hulbert et al. 1990) and subsequent work had shown conserved synteny over even longer evolutionary timeframes leading to the model of concentric circle genomes based on conserved marker order among a number grasses (Moore et al. 1995).

Even though considerable genetic synteny was found in comparisons of other cereals to rice, sequencing of orthologous genomic regions uncovered considerable variation, in gene content, repeat structure and size. One of the first comparisons of rice, maize and sorghum, preceding the rice genome sequencing project, revealed extensive size variation, especially inflated in maize, and in conservation of low copy sequences among the three species (Chen et al. 1997). Subsequent studies have shown that while rice is generally predictive of gene content in related cereal genomes, many genes are missed as either absent in rice, or present in rice and absent in the comparator. For instance in a rice-barley comparison around a disease resistance locus, barley had six additional genes not found in the rice orthologous region (Moore et al. 1995). Thus, while useful for leveraging genetics in other cereal species, additional crop genome sequences were required to fully address the genetic underpinning of traits in those crops.

### Contribution to Advancement of Rice Genetics and Improvement

#### Gene and QTL Cloning

**Fig. 1:**
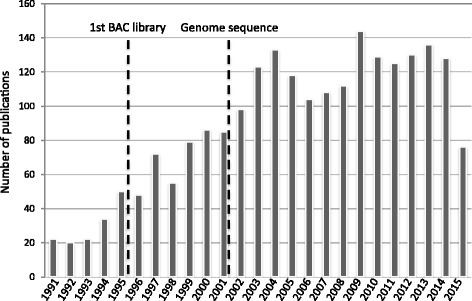
Publications related to gene cloning in rice. ISI Web of Science (26-August-2015): search ‘rice’ and ‘gene cloning’ 1990–2015, retrieved *2,237 records*

Gene cloning and especially cloning genes underlying QTL can provide a deeper molecular understanding of a trait and in the case of breeding, markers linked directly with the causative DNA mutation/changes. In rice, from 1990 to 1994 there were 20–30 papers per year retrieved by using ‘gene cloning’ as a search term (Fig. 1). In 1995, the first rice BAC library was published (Wang et al. 1995) which was eventually used for cloning of the Xa-21 resistance gene (Song et al. 1995). Between 1995 and 2001, as more cloning tools became available, the number per year ranged in the 40–80. After the genome sequence, the number jumped to more than 100 per year and has been fairly stable since. Even though this analysis is very rough, no manual curation of the extracted references, just a count of returned citations, it seems obvious that the genomic resources of large insert DNA clones and eventually the genome sequence greatly advanced gene/QTL cloning in rice. An obvious example of where the genome facilitated cloning of a gene underlying a QTL is *SUB1*, the gene that confers subermergence tolerance (Xu et al. 2006). Another outgrowth of the reference genome is the exploration of diversity at a genome scale across the entire Oryza genus (Ammiraju et al. 2008; Lu et al. 2009; Sanyal et al. 2010; Jacquemin et al. 2014), including exploration within relatives closely related to cultivated rice (AA genome species) (Wang et al. 2014; Zhang et al. 2014). Many of these explorations depended on resources that arose from the International Oryza Map Alignment Project (Wing et al. 2005; Ammiraju et al. 2006).

### Contribution of the Rice Genome to Improvement

The bigger goal for the community is rice improvement. On several fronts the rice reference genome has greatly advance rice improvement. First, the most immediate impact was in molecular markers in that the number was greatly increased, their physical order was understood and proximity to annotated genes was useful to predict gene-trait associations. In part, this has been driven by reduction in DNA sequencing costs that have allowed researchers to resequence additional rice accessions and call nucleotide variations relative to the reference genome (McNally et al. 2009). Second, and related to the first, in-depth, sequence-based analysis of variation in cultivated and wild rice to allow breeders to better understand and exploit genetic variation, as proposed by McCouch and colleagues (McCouch et al. 2012). Third, molecular understanding of the genetic basis of traits such as N and P-use is allowing rice researchers to engineer ‘Green Super Rice’ that should help meet the challenge of the growing world population while requiring fewer inputs (Zhang 2007).

## Conclusions

The availability of the rice genome, together with the community annotation and other resources that added functionality, transformed genetics research and rice breeding. This can be measured any number of ways, but the increase in gene/QTL cloning is one direct measure. Ongoing work to understand the evolution and domestication of rice and to use this and information on the genetic architecture underlying plant physiological responses and phenotypes is now be used to engineer superior rice strains to feed our growing population.

## Abbreviations

BAC: bacterial artificial chromosome
bp: base pair
QTL: quantitative trait loci
RFLP: restriction fragment length polymorphisms
